# A Hybrid In Silico and Tumor-on-a-Chip Approach to Model Targeted Protein Behavior in 3D Microenvironments

**DOI:** 10.3390/cancers13102461

**Published:** 2021-05-18

**Authors:** Valentina Palacio-Castañeda, Simon Dumas, Philipp Albrecht, Thijmen J. Wijgers, Stéphanie Descroix, Wouter P. R. Verdurmen

**Affiliations:** 1 Department of Biochemistry, Radboud Institute for Molecular Life Sciences (RIMLS), Radboud University Medical Center, Geert Grooteplein 28, 6525 GA Nijmegen, The Netherlands; Valentina.Palacio-Castaneda@radboudumc.nl (V.P.-C.); philipp.albrecht@uni-jena.de (P.A.); t.janwijgers@gmail.com (T.J.W.); 2 Physico-Chemistry Curie, Institut Curie, PSL Research University, CNRS UMR168, Sorbonne University, 75005 Paris, France; simon.dumas@curie.fr (S.D.); stephanie.descroix@curie.fr (S.D.)

**Keywords:** tumor-on-a-chip, spheroid, DARPin, protein diffusion, tumor targeting

## Abstract

**Simple Summary:**

Engineered proteins possess a great therapeutic potential, but the development of such therapies is impeded during preclinical studies by the lack of in vitro models that accurately simulate the human physiology. Animal models, on the other hand, also have difficulties predicting human responses, and are ethically concerning. In this study, we employed a hybrid approach where we combined mathematical modeling with 3D in vitro models that mimic aspects of the tumor microenvironment, in order to simulate the delivery of therapeutic proteins targeting cancer cells and to predict the biological activity. By cross-comparing simulated and experimental data from 3D models, we were able to correctly predict the best dose needed to deliver toxic proteins specifically to tumor cells, while leaving the surrounding non-tumor cells untouched. This study shows the potential of combining computational approaches with novel in vitro models to advance the development of protein therapeutics.

**Abstract:**

To rationally improve targeted drug delivery to tumor cells, new methods combining in silico and physiologically relevant in vitro models are needed. This study combines mathematical modeling with 3D in vitro co-culture models to study the delivery of engineered proteins, called designed ankyrin repeat proteins (DARPins), in biomimetic tumor microenvironments containing fibroblasts and tumor cells overexpressing epithelial cell adhesion molecule (EpCAM) or human epithelial growth factor receptor (HER2). In multicellular tumor spheroids, we observed strong binding-site barriers in combination with low apparent diffusion coefficients of 1 µm^2^·s^−1^ and 2 µm^2^ ·s^−1^ for EpCAM- and HER2-binding DARPin, respectively. Contrasting this, in a tumor-on-a-chip model for investigating delivery in real-time, transport was characterized by hindered diffusion as a consequence of the lower local tumor cell density. Finally, simulations of the diffusion of an EpCAM-targeting DARPin fused to a fragment of *Pseudomonas aeruginosa* exotoxin A, which specifically kills tumor cells while leaving fibroblasts untouched, correctly predicted the need for concentrations of 10 nM or higher for extensive tumor cell killing on-chip, whereas in 2D models picomolar concentrations were sufficient. These results illustrate the power of combining in vitro models with mathematical modeling to study and predict the protein activity in complex 3D models.

## 1. Introduction

Engineered proteins including antibodies and non-immunoglobulin binding proteins that target cancer-specific features play an increasingly important role in the development of novel cancer therapies [1]. Compared with small molecules, proteins offer enhanced specificity and/or selective recognition of surface features [2]. Furthermore, their small size offers benefits with respect to tissue accessibility compared with most nanoparticulate delivery systems [3]. Nevertheless, the efficient delivery of therapeutic proteins into tumors can be hindered because of a binding-site barrier [4], a high interstitial pressure as a result of poor tumor vasculature, a defective lymphatic system, and a dense extracellular matrix [5]. When the local concentration of the therapeutic agent is sublethal deep inside the tumor tissue, not only is a less efficacious treatment observed, but the development of therapy resistance is also much more likely [6,7].

Traditionally, tumor targeting has been studied using 2D culture models and animal models. However, the aforementioned barriers to efficient drug delivery are not replicated in 2D culture systems, and in vivo models suffer from a low throughput. Furthermore, in vivo models are difficult to control, expensive, less suitable for real-time uptake studies, and ethically problematic. In addition, there are increasing worries regarding the poor extrapolation of results from animal model outcomes to the human setting [8,9,10]. Consequently, there has been growing interest in the utilization of in vitro complex 3D tissues that mimic relevant aspects of human tumor microenvironments for studying drug delivery [11,12].

Multicellular tumor spheroids (MCTS) consisting of a cluster of tumor cells are a suitable 3D model to study drug delivery to avascular tumor regions [12]. MCTS are one of the simplest 3D models that can replicate tumor features, such as gradients of oxygen and waste products and the high density of the extracellular matrix [12,13]. MCTS can be generated with a high throughput and have been used to efficiently study the penetration of nanoparticles, antibodies, and antibody fragments in 3D [14,15,16]. Alternative models that include more complex features of the tumor microenvironment have gained more attention in recent years. In particular, microfluidic cancer-on-a-chip platforms, either consisting solely of cancer cells or co-cultured with stromal cells [17,18,19,20,21], provide a physiologically more relevant 3D microenvironment because of the ability to compartmentalize and better control gradients [22,23]. Furthermore, cancer-on-a-chip systems also allow for real-time imaging and quantitative analyses of the biological processes in ways unachievable with current animal models. Therefore, these models have the potential for a more in-depth investigation of the transport processes of drugs, including therapeutic proteins, into and around tumor tissues, and allow for a quick evaluation of novel formats with respect to their transport behavior. Importantly, the highly controlled nature of experiments with microfluidic models facilitates the application of modeling approaches for the description of transport processes, which, so far, have been limited to the description of processes in either static spheroids [14] or in vivo models [24,25,26,27,28].

**Figure 1:**
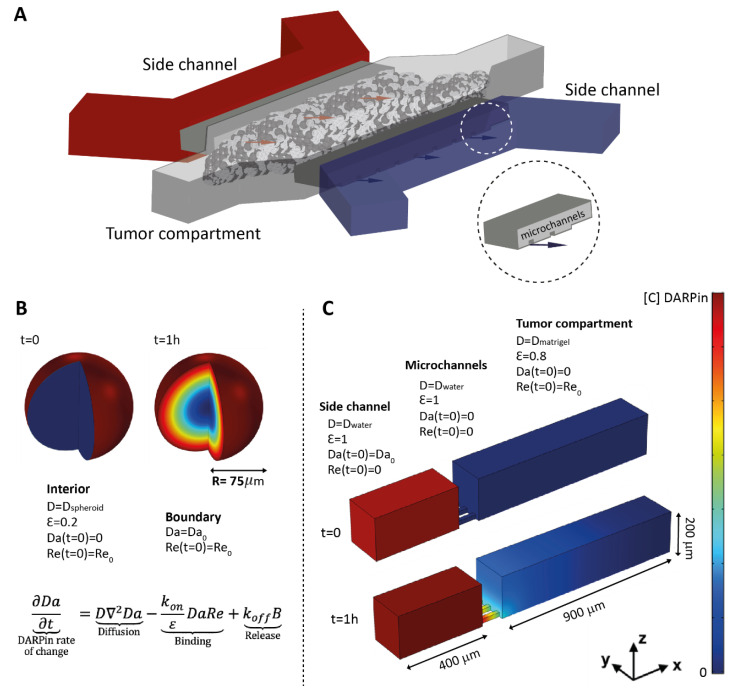
Overview of the tumor-on-a-chip system and conditions for the mathematical model in spheroids and on-chip. (**A**) Illustration of the microfluidic tumor-on-a-chip system showing the side channels used for DARPin delivery towards the tumor channel, where tumor cells or tumor cells and fibroblasts together grow embedded in Matrigel. The insert shows a close-up view of the microchannels that connect the side channels of the tumor compartment. (**B**) Spheroids were modelled in COMSOL as spheres into which the free DARPins (Da) diffused from outside to inside, while encountering receptors on the surface of the cells (Re). The model was simplified into a 1D model using the spherical expression of Equation (1) (see Material and Methods). B refers to DARPin–receptor complex. (**C**) The tumor-on-a-chip 3D geometry was reproduced in COMSOL. DARPins diffuse from the side channel to the tumor compartment, which is connected by small microchannels. The matrix properties and cell density were taken into account by implementing distinct diffusion coefficients (abbreviated as D_water_ and D_matrigel_) and available volume (void) fractions (ε) for the tumor compartment and for the side channel.

In this study, we investigated the specific delivery of designed ankyrin repeat proteins (DARPins) directed against human epidermal growth factor receptor 2 (HER2) or epithelial cell adhesion molecule (EpCAM) in complex 3D tumor microenvironments. We included optically cleared spheroids and a microfluidic tumor model for real-time visualization of the tumor penetration, using 2D models as a reference. Based on our experimental setup, we also developed a mathematical model for evaluating protein delivery in spheroids and on-chip (Figure 1). We used DARPins because they are highly stable engineered proteins that can be selected to bind, with very high affinities, to virtually any target [29]. Additionally, DARPins are 10× smaller than conventional antibodies, giving them better tissue-penetration properties [30]. To investigate the specific activity towards tumor cells in our tumor-on-a-chip system, we fused DARPins to the *Pseudomonas aeruginosa* exotoxin A (ETA) and compared the experimental data with mathematical predictions of an adapted mathematical model considering the cell-killing activity of the agents. The predictions of the model aligned with our experimental observations regarding the expected amount of cell death throughout the microfluidic chip after 48 h, illustrating the potential of a hybrid approach that combines tumor-on-a-chip systems with mathematical models for investigating the transport processes and biological activities of candidate protein therapies.

## 2. Materials and Methods

### 2.1. Cell Lines and Culture Media

The experiments were performed with human lung adenocarcinoma Calu-3 cells, human mammary gland ductal carcinoma BT-474 cells (both ATCC, Manassas, VA, USA), and two primary human fibroblast cell lines—IMR-90 (ATCC, Manassas, VA, USA ) and C5120—which were kindly donated by Dr. Rodenburg (Radboud University Medical Center, Nijmegen, The Netherlands) [31]. The BT-474 cells were cultured in Gibco Roswell Park Memorial Institute medium (RPMI), Calu-3 cells in a minimum essential medium Eagle (MEM), and the fibroblasts in Medium 199 (Gibco, Invitrogen, Carlsbad, CA, USA). All of the media were supplemented with 10% fetal calf serum (FCS). For the experiments with microfluidic devices, 1% *v*/*v* penicillin-streptomycin (Sigma-Aldrich, St. Louis, MO, USA) and 2.5 µg mL^−1^ Amphotericin B (Sigma-Aldrich, St. Louis, MO, USA) were supplemented to the media.

### 2.2. Cloning, Expression and Conjugation of DARPins and DARPin Fusions

**Figure 2:**
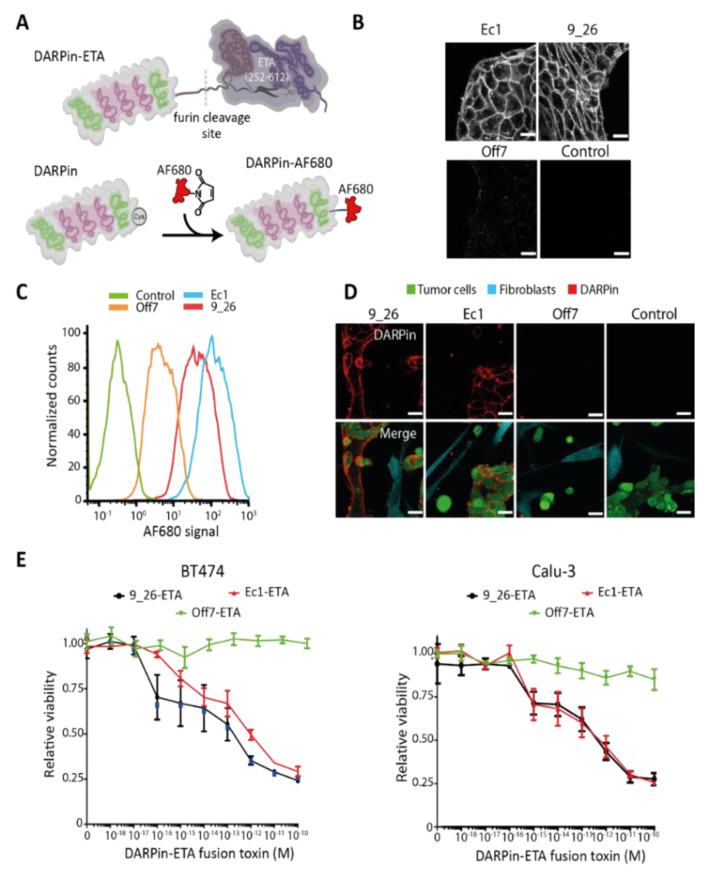
Binding and specificity of DARPins targeting EpCAM or HER2 on different cancer cells. (**A**) Schematic representation of a DARPin fused to the translocation and catalytic domains of *Pseudomonas aeruginosa* exotoxin A (ETA; aa 252–612) and of a DARPin coupled to Alexa Fluor 680. (**B**) Confocal microscopy images of an Alexa Fluor-680-labeled EpCAM-binding DARPin (Ec1) and a HER2-binding DARPin (9_26) bound to live Calu-3 cells. Off7 was included as a non-binding control. Scale bars: 40 µm. (**C**) Representative flow cytometry histogram showing the binding of Alexa Fluor (AF) 680-labeled DARPins to Calu-3 cells, *n* = 2. (**D**) Specificity of DARPins towards Calu-3 cells in co-cultures with IMR-90 fibroblasts. Scale bars: 40 µm. (**E**) Toxicity in 2D of BT-474 and Calu-3 cells of DARPin-ETA fusion proteins after 48 h of incubation as evaluated by a resazurin assay.

The EpCAM-binding DARPin Ec1 and the control DARPin Off7 with an N-terminal MRGS-His6 tag and a C-terminal cysteine and FLAG tag were expressed from pQIq vectors in the *Escherichia coli* (*E. coli*) strain BLR(DE3), as described previously [32]. The HER2-binding DARPins 9_26 was similarly cloned in pQIq through BamHI and HindIII restriction sites and was expressed in BLR(DE3). The DARPins fused to the translocation and catalytic domains of *Pseudomonas aeruginosa* exotoxin A (ETA; aa 252–612) were generated by replacing the consensus DARPin NI_3_C in Ec1-ETA (252-412)-NI_3_C-KDEL with the catalytic domain of ETA through SpeI and PstI restriction sites [33]. The EpCAM-binding DARPin Ec1 was subsequently replaced by the HER2-targeting DARPins 9_26, or the control DARPin Off7 through the BamHI and HindIII restriction sites. The DARPin-fusion proteins were expressed in the *E. coli* strain BL21(DE3) essentially, as described previously [33], with the exception that a 2YT medium (16 g·L^−1^ peptone, 10 g·L^−1^ yeast extract, 5 g·L^−1^ NaCl) containing 50 μg mL^−1^ ampicillin (Sigma-Aldrich, St. Louis, MO, USA) was used for the protein expression. DARPins were coupled to Alexa Fluor 680 (Thermo Fisher Scientific, Waltham, MA, USA), as described previously [32]. The purified DARPins and DARPin-toxin fusions are shown in Figure S1A,B, and a schematic of the engineered DARPins is presented in Figure 2A, as well as the confirmation of the specific binding to Calu-3 cells in Figure 2B.

### 2.3. Quantification of Receptor Density

The cells were detached using a 10 mM EDTA solution (Merck, Darmstadt, Germany) at 4 °C and were stained with a Phycoerythrin (PE)-labeled anti-EpCAM antibody (Abcam, Cambridge, MA, USA) or PE-labeled anti-HER2 antibody (Abcam, Cambridge, MA, USA). A PE-labeled IgG1 isotype control antibody (Life Technologies Carlsbad, CA, USA) was used as a negative control. The fluorescence intensity for each antibody was measured on a MACSQuant Analyzer 10 flow cytometer (Miltenyi, Bergisch Gladbach, Germany), and a calibration curve was generated using the BD QuantiBrite™ PE fluorescence quantitation kit (BD Biosciences, Franklin Lakes, NJ, USA), according to the manufacturer’s instructions. The data were analyzed with FlowJo 10.0.8rl (BD Life Sciences, Ashland, OR, USA). For quantifying the receptor number (Figure S2), we utilized the median of the whole cell population for EpCAM, whereas for the quantification of HER2 receptor density, we used the median of the positive cell population because of inhomogeneous staining. Through staining with receptor-targeting DARPins, we could confirm the homogeneous expression of HER2 on the cell lines (Calu-3: Figure 2C, BT-474: Figure S3), which led us to conclude that staining with the commercial antibodies was incomplete. Staining with DARPins also supported the similar number of binding sites found for EpCAM and HER2 on BT-474 cells (Figure S3).

### 2.4. Determination of Binding of DARPin to Cell Surface Receptors

Calu-3, BT-474, and IMR-90 cells were seeded in 24-well culture plates. The cells were grown overnight and subsequently incubated for 2 h with Alexa Fluor 680-labeled anti-EpCAM or anti-HER2 DARPins at 37 °C. The cells were then washed twice with PBS and detached with PBS containing 5 mM EDTA for Calu-3 and BT-474. The IMR-90 cells required detachment with trypsin. The cells were centrifuged at 300× *g* for 3 min and resuspended in PBS. The cells were measured using the MACSQuant Analyzer 10 flow cytometer (Miltenyi, Bergisch Gladbach, Germany), and the data were analyzed with FlowJo 10.0.8rl (BD Life Sciences, Ashland, OR, USA).

### 2.5. Specific DARPin Binding to Tumor Cells in Co-Cultures

Calu-3 cells and IMR-90 cells were stained with carboxyfluorescein succinimidyl ester (CFSE) or CellTrace yellow (both from Thermo Fisher Scientific, Waltham, MA, USA), respectively. The cells were seeded at a density of 50,000 cells/well for Calu-3 and 30,000 cells/well for IMR-90 in µ-slide eight-well chambers (Ibidi, Gräfelfing, Germany). Co-cultures were grown overnight and subsequently incubated for 2 h with various DARPin-Alexa Fluor 680 conjugates at 500 nM for 2 h. The cells were washed with complete medium (Medium 199, Life Technologies Carlsbad, CA, USA) and images were taken with an HC PL APO 63x/1.20 N.A. water immersion objective on a Leica TCS SP8 confocal microscope (Leica Microsystems, Wetzlar, Germany).

### 2.6. Toxicity of DARPin-Toxin Fusions

Calu-3 cells were seeded in a 96-well plate at a density of 20,000 cells/well. The cells were grown overnight and incubated for 48 h with DARPins fused to the translocation and catalytic domains of ETA. The cells were then either incubated with a 0.1 mg·mL^−1^ resazurin working solution in complete medium and incubated for 2 h, or grown for 96 h more before being incubated with resazurin. Afterwards, fluorescence was measured with 525–540 nm excitation and 620–640 nm emission light on a BioTek Synergy 2 Multi-Mode Reader (BioTek Instruments, Winooski, VT, USA). The relative viability was calculated from the fluorescent signals by subtracting the resazurin background signal and normalizing to untreated cells.

### 2.7. DARPin Penetration into Tumor Spheroids

BT-474 spheroids were produced via the hanging-drop method by pipetting drops of 30 μL with 5000 cells, as described previously [20]. Spheroids were allowed to form overnight at 37 °C in an incubator with 5% CO_2_. The DARPins were added in 1.5 μL to the 30 μL drops with the spheroid and were incubated for the indicated times at 37 °C. Spheroids were fixated for 2 h in 4% (*w/v*) PFA (Sigma-Aldrich, St. Louis, MO, USA) and kept in 2% PFA overnight at 4 °C. The spheroids were washed and embedded in a 20 μL drop of collagen solution (4 mg·mL^−1^) in an eight-well IBIDI chamber (Gräfelfing, Germany). The IBIDI chamber was kept at room temperature for 5 min, after which the collagen was polymerized for 40 min at 37 °C. A modified SeeDB protocol was employed for clearing, as described previously [20]. Confocal images were taken with an HCX PL APO 40x/0.85 dry objective or an HCX PL APO 10x/0.40 dry objective on a Leica TCS SP8 confocal microscope (Leica Microsystems, Wetzlar, Germany).

### 2.8. Quantification of DARPin Penetration Depth in Tumor Spheroids

A python-based macro for Fiji [34] was created and used to quantify the penetration depth of the fluorescently labeled DARPins into the BT-474 spheroids (Supplementary Methods). The slice representing the widest cross-section, and hence the maximum distance from spheroid rim to its center, was used for the quantification of penetration. First, the region of interest (ROI) was manually selected around the spheroid. After determining the ROI center, 360 radial lines covering 360° were drawn from the center to the border of the ROI. Next, a Gaussian blur was applied before determining the signal intensity along the lines. The maximum intensity values determined for 360 lines were averaged. The half-maximal intensity was calculated, which was set as the rim of the spheroid, and was used to determine the distance of penetration. To determine the spheroid size, the radii were averaged. Tumor penetration was expressed as a percentage of the relative penetration from the outer rim of the spheroid.

### 2.9. Fabrication of Tumor-on-a-Chip

A design similar to the one reported by Chernyavska et al. was employed, composed of three parallel channels on glass on a device fabricated utilizing replica-molded polydimethylsiloxane (PDMS; Dow Corning, Midland, MI, USA) [20]. In this case, the spheroid traps were removed from the central chamber and 5 mm holes were punched to make the reservoirs connected to the side channels, using a biopsy puncher (Kai Industries, Seki, Gifu, Japan). All of the manufacturing steps were performed as reported previously [20].

### 2.10. Real-Time DARPin Penetration in the Tumor-on-a-Chip System

Co-cultures of IMR-90 or C5120 fibroblasts were labeled according to the manufacturer’s instructions with CellTrace yellow, and CellTrace violet was used for the BT-474 cells. The labeled cells were then embedded in a 10 mg·mL^−1^ Matrigel (Corning, Corning, NY, USA) matrix and loaded manually in the tumor compartment of the microfluidic chip at a density of 2.0 × 10^7^ cells mL^−1^. The chips were placed in the incubator at 37 °C) for 15 min to allow the Matrigel to polymerize. After this time, a medium was added in the side channels and each of the four reservoirs were filled with the same volume of medium. We compared the Matrigel with collagen (4 mg·mL^−1^), but under the evaluated circumstances, we noted significant contraction of the collagen gel. After 48 h, protein diffusion through the Matrigel matrix and the specific targeting of cancer cells was investigated by adding Alexa Fluor 680-labeled DARPins in one of the side channels and filling up the two reservoirs with the exact same volume of medium immediately before imaging. Using the LASX software (Leica Microsystems, Wetzlar, Germany) with a Leica TCS SP8 confocal microscope (Leica Microsystems, Wetzlar, Germany) equipped with an HCX PL APO 10x/0.40 dry objective (Leica Microsystems, Wetzlar, Germany), one position per chip was selected and time lapses were recorded by taking an image of each marked position every 1 min for 1 h. For the image analyses and subsequent comparisons with the simulated data, Fiji was used. The Matrigel region of the middle channel was manually selected and plot profiles of fluorescence intensity across this channel were generated for the images at 0, 15, 30, 45, and 60 min. The intensity values were converted to concentrations using an averaged background-corrected maximum value in the side channel and the concentration at which DARPins were added (500 nM). We assumed a linear relationship between the fluorescence intensity and concentration.

For the toxicity studies in the tumor-on-a-chip, BT-474 cells labeled with CFSE and C5120 fibroblasts labeled with CellTrace violet were grown in the chip for 24 h. After this, DARPin-ETA fusions were added to one of the side channels of the chip at concentrations of 1 or 10 nM. The cell viability after 48 h of culture was determined by adding the cell death staining propidium iodide (PI; Sigma-Aldrich, St. Louis, MO, USA) into the chips and through quantification, as described below.

### 2.11. Quantification of Cell Death in Tumor-on-a-Chip System

To quantify the relative amount of cell death of each cell type in the tumor-on-a-chip after treatment with the DARPin-toxin fusions, a macro in Fiji was developed (Supplementary Methods). Briefly, each channel was filtered using a Gaussian blur. A threshold was applied to the images and the positive area corresponding to the cells or the PI staining was converted into a region of interest (ROI). The ROIs corresponding to one cell channel and PI were overlapped, and a ROI of the overlapping areas was created. Subsequently, the percentage of the overlapping area compared to the total cell area in the threshold image was calculated. The relative amount of cell death (based on surface area) was then reported as the ratio of the overlapping area between the PI channel and the cell channel.

### 2.12. Confocal Microscopy Imaging Settings

CellTrace Violet was excited at 405 nm (detection: 413–460 nm), CFSE at 492 nm (detection: 500–520 nm), CellTrace Yellow at 546 nm (detection: 554–580 nm), and CytoTell Red at 647 nm (detection: 656–750 nm). The Alexa Fluor 680-labeled DARPins were excited at 679 nm (detection: 688–720 nm) and propidium iodide at 535 nm (detection: 543–620 nm).

### 2.13. Mathematical Model of DARPins Diffusion In Vitro

A binding-diffusion mathematical model was implemented by introducing the custom governing equations (Equations (1) to (3)) in the finite element software COMSOL Multiphysics v5.5 (Stockholm, Sweden) using the equation-based interface. A schematic of the input variables related to the therapy and the tissue, as well as the modelled outcomes, is given in Figure S4. The underlying coupled equations were initially reported by Thurber et al. [14] and Torres et al. [28], and describe the kinetics of unbound DARPin (1), receptor (2), and DARPin–receptor complex (3), respectively.

(1) $$\underset{\begin{array}{c} \mathrm{DARPin}\;\mathrm{rate}\\ \mathrm{of}\;\mathrm{change} \end{array}}{\underbrace{\frac{\partial \left[Da\right]}{\partial t}}}=\underset{\mathrm{Diffusion}}{\underbrace{D\nabla^{2}\left[Da\right]}}-\underset{\mathrm{Binding}}{\underbrace{\frac{k_{on}}{\varepsilon }\left[Da\right]\left[Re\right]}}+\underset{\mathrm{Release}}{\underbrace{k_{off}\left[B\right]}}$$

**Equation (1).** DARPin.

(2) $$\underset{\begin{array}{c} \mathrm{Receptor}\;\mathrm{rate}\\ \mathrm{of}\;\mathrm{change} \end{array}}{\underbrace{\frac{\partial \left[Re\right]}{\partial t}}}=\underset{\begin{array}{c} \mathrm{Synthesis}\;\mathrm{of}\\ \mathrm{new}\;\mathrm{receptor} \end{array}}{\underbrace{R_{s}}}-\underset{\mathrm{Binding}}{\underbrace{\frac{k_{on}}{\varepsilon }\left[Da\right]\left[Re\right]}}+\underset{\mathrm{Release}}{\underbrace{k_{off}\left[B\right]}-}\underset{\mathrm{Endocytosis}}{\underbrace{k_{e}\left[Re\right]}}$$

**Equation (2).** Receptor.

(3) $$\underset{\begin{array}{c} \mathrm{Complex}\;\mathrm{rate}\\ \mathrm{of}\;\mathrm{change} \end{array}}{\underbrace{\frac{\partial \left[B\right]}{\partial t}}}=\underset{\mathrm{Binding}}{\underbrace{\frac{k_{on}}{\varepsilon }\left[Da\right]\left[Re\right]}}-\underset{\mathrm{Release}}{\underbrace{k_{off}\left[B\right]}-}\underset{\mathrm{Endocytosis}}{\underbrace{k_{e}\left[B\right]}}$$

**Equation (3).** DARPin—receptor complex.

Where Da, Re, and B are the DARPin, receptor, and DARPin–receptor complex concentrations, respectively. The starting value of Da in the side channel reflects the chosen concentration and is indicated per experiment. The values for Re were experimentally determined or taken from the literature (Table 1). The starting value of B is, by definition, zero.

D is the DARPin diffusion coefficient in ECM and was determined experimentally (Figure S5A); ε is the interstitial volume fraction (see below for how values were acquired); and k_on_, k_off_, and k_e_ are the binding, release, and endocytosis rate constants of the receptors, respectively and were taken from the literature (Table 1). Re_0_ is the receptor concentration at t = 0. Note that the binding terms k_on_ and k_off_ must be divided by the available interstitial volume fraction for the interstitial diffusion, referred to as the void fraction (ε), because the DARPin–receptor interactions only occur in the space accessible to DARPins. ε is calculated as follows, ε = V_ECM/V_total, where V refers to volume and ECM to the extracellular matrix.

We considered the cells to be immobile, so that among the three variables, only Da can diffuse (D not equal to zero). The following two distinct assumptions could be made for the receptor regeneration term of R_s_: (i) If cells are not killed during DARPin diffusion, all receptors are recycled, R_s_ = k_e_ (Re + B). This assumption was taken when investigating the delivery of non-toxic DARPins. (ii) If cells are killed, internalized DARPin–receptor complexes are no longer recycled, R_s_ = k_e_Re. This assumption was taken when investigating the delivery of fusion toxins, i.e., DARPins fused to a fragment of the bacterial toxin ETA.

Transient simulations were performed to predict the concentration profiles over time in both spheroids and tumor-on-chip systems.

Diffusion inside the spheroids was modelled on a spherical geometry, as shown in Figure 1A. The model was simplified into a 1D problem by taking the spherical coordinates’ expression for the Laplace operator. For the boundary conditions, a constant surrounding the DARPin concentration was assumed at the sphere border, and zero-flux was assumed at the center (r = 0), so as to consider the spherical symmetry. The void fraction for modeling the delivery in spheroids was taken from the literature (Table 1).

The tumor-on-chip model was solved on a 3D geometry, as shown in Figure 1B. To consider the presence of cells in the matrix, distinct values for the void fraction and diffusion coefficient (D_water_ and D_ECM_) were used for the tumor compartment and the side channel. The void fraction for modeling the delivery in the tumor-on-a-chip model was estimated by subtracting the volume occupied by CellTrace yellow-stained BT–474 cells in a Z-stack from top to bottom in the central tumor compartment from the total measured tumor compartment volume in the same Z-stack. The side channels are connected to the middle compartment by smaller channels that are 100 µm long, 20 µm × 20 µm wide, and 40 µm spaced. All of the boundaries were defined with a zero-flux condition, except for the side walls (normal to *y*-axis), which were set as symmetric. The initial conditions are shown in Figure 1B.

To determine the diffusion coefficient of DARPins in the ECM for the tumor-on-chip model, we performed simulations for the non-binding Off7 DARPin with different values for the diffusion coefficient. We then chose the value that provided the best fitting with experiments through a least-squares minimization method (Figure S5A). The obtained value, of 55 µm^2^·s^−1^, was kept for the subsequent simulations with binding DARPins, assuming a comparable hydrodynamic radius for each.

## 3. Results

### 3.1. Specificity of DARPins and Toxicity of DARPin-Toxin Fusion Proteins in 2D Model Systems

As a reference point for the evaluation in 3D tumor models, we started by investigating the binding specificity of DARPins and the cell-selective toxicity of DARPin-toxin fusions in flat 2D monolayer systems. We used DARPin 9_26, which binds to HER2 with a a dissociation constant (K_d_) of 1.4 nM [35], and EpCAM-binding DARPin Ec1, which binds to EpCAM with a K_d_ of 68 pM [36], including the non-binding control Off7 (Figure S1A). DARPins were produced with a unique cysteine for fluorescent labeling or were fused to a fragment of ETA consisting of the translocation domain and the catalytic domain (Figure 2A and Figure S1B). DARPin-toxin fusions are highly potent receptor-targeted fusion toxins [39].

We used the human breast carcinoma cell line BT-474 and the human lung carcinoma cell line Calu-3 as model cancer cell lines, which both overexpress HER2 and EpCAM [40,41,42]. The number of EpCAM and HER2 molecules on the cell surface of both cell lines was quantified using flow cytometry. An average of 3.86 × 10^5^ ± 4.8 × 10^4^ EpCAM molecules/cell was quantified for BT-474 and 2.58 × 10^5^ ± 9.9 × 10^3^ EpCAM molecules/cell for Calu-3 (Figure S2A). For HER2, the expression on BT-474 and Calu-3 was found to be 3.15 × 10^5^ (range: 3.04–3.27 × 10^5^) molecules/cell and 1.59 × 10^5^ (range: 1.54–1.62 × 10^5^) molecules/cell, respectively (Figure S2B). Primary lung or skin fibroblasts (IMR-90 and C5120, respectively) were included in order to investigate the cancer cell specificity. C5120 is reported to be low on EpCAM [20] and HER2 [31], and we found the same for IMR-90 (Figure S2A,B).

HER2-binding 9_26 and the EpCAM-binding DARPin Ec1 showed a clear membrane staining and some vesicular uptake when incubated with Calu-3 cells (Figure 2B). The control DARPin Off7 showed very low non-specific signals. The specific binding of the DARPins was further quantified by flow cytometry (Figure 2C and Figure S3), which confirmed the highly specific binding of Ec1 and 9_26, but not of the control Off7. In co-cultures of IMR-90 fibroblasts and Calu-3 tumor cells, a specific binding of Ec1 and 9_26 to tumor cells was observed (Figure 2D). No enrichment of the non-binding control DARPin on the tumor cells was seen.

Additionally, we evaluated the toxicity towards the cancer cell lines of DARPin-toxin fusions containing either 9_26, Ec1, or Off7 after 48 h (Figure 2E). We observed a 75% reduction in cell viability for both targeted fusion toxins, already at subnanomolar concentrations. To confirm that the residual viability was due to the short incubation period, we incubated cells for 48 h with fusions toxins, washed away the fusion toxins, and incubated them for an additional 72 h. At that time point, the targeted toxin mediated 100% tumor cell killing already at 10 pM, whereas the control fusion toxin showed no toxicity (Figure S6).

### 3.2. DARPin Delivery in 3D Multicellular Tumor Spheroids (MCTS)

**Figure 3:**
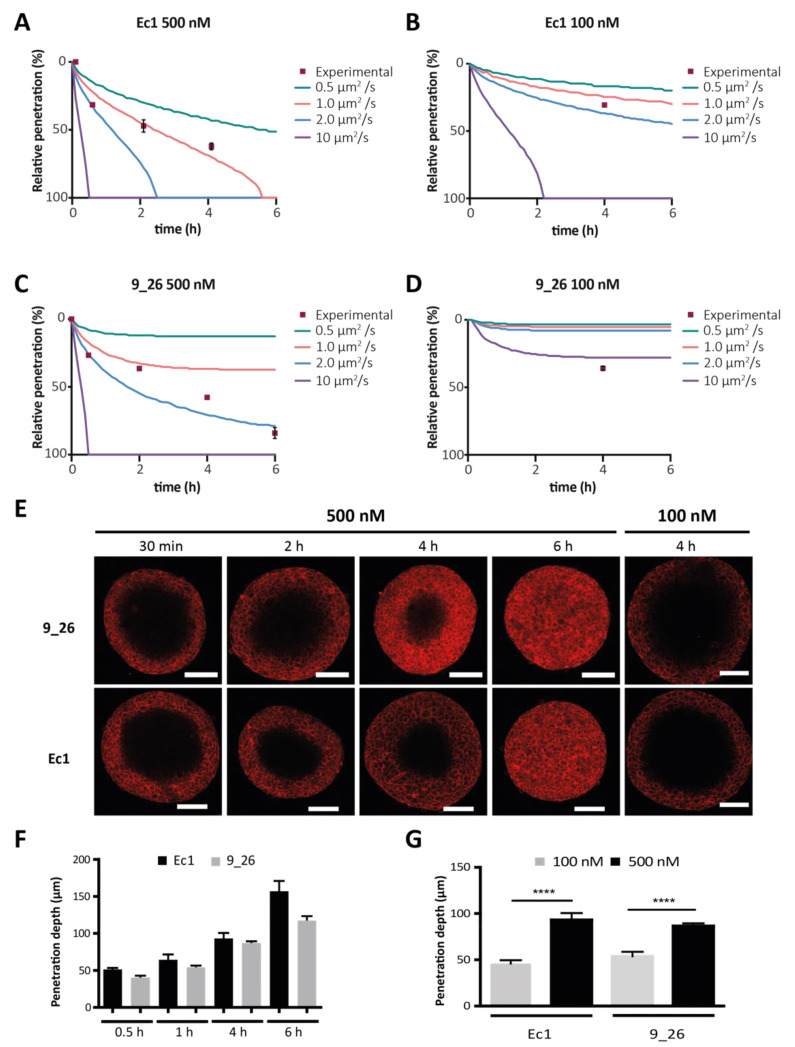
Simulation and experimental validation of DARPin penetration into tumor spheroids. (**A**–**D**) Experimental data vs. simulation data using four different diffusion coefficients. The *y*-axis is the percentage of penetration (i.e., of total distance) from the outside towards the core. The spheroid radius was set at 150 µm for simulated data, which was the average radius; *n* = 5 spheroids per time point. The distinct conditions were Ec1 at 500 nM (**A**), Ec1 at 100 nM (**B**), 9_26 at 500 nM (**C**) and 9_26 at 100 nM (**D**). (**E**) Confocal microscopy images of cleared BT-474 spheroids at several time points of incubation of DARPins at 500 nM or after 4 h incubation at 100 nM. Scale bars are 100 µm. (**F**) Quantification of the time dependency of the penetration depth of DARPins Ec1 and 9_26 in BT-474 spheroids; *n* = 5. (**G**) Quantification of concentration dependency of DARPin penetration into BT-474 spheroids; *n* = 3, error bars correspond the standard error of the mean (SEM). **** *p* < 0.0001 based on a two-way ANOVA.

To evaluate the potential of mathematical modeling to predict DARPin diffusion in multicellular tumor spheroids (MCTS), we simulated DARPin delivery using a mathematical model similar to the one reported by Torres et al. for the investigation of in vivo protein delivery [28]. MCTS are a reliable 3D in vitro model for simulating the features of avascular tumors, including a binding-site barrier, because of a high receptor density. Optical clearing allows for the visualization of fluorescence throughout the entire spheroid using confocal microscopy [20,32]. Given our knowledge on most of the quantifiable parameters determining tumor penetration (Table 1), the unknown variable in our model for fitting variation in the speed of penetration is the apparent diffusion coefficient. We refer to this value as the apparent diffusion coefficient, because it takes into account the tortuosity of diffusion in the spheroid and is thus lower than the true diffusion. We simulated the tumor penetration of Ec1 and 9_26 at 500 nM and 100 nM in BT-474 spheroids with diffusion coefficients ranging between 0.5 to 10 µm^2^·s^−1^. For Ec1 at 500 nM, the simulations yielded a full penetration after ~5.5 h with an estimated diffusion coefficient of 1.0 µm^2^·s^−1^ (Table 1), which agreed with the experimental data, and at 100 nM, a full penetration of the spheroids would occur after ~2 h at a diffusion coefficient of 10 µm^2^·s^−1^ (Figure 3B). For 9_26 at 500 nM, the model predicted that, contrary to Ec1, even with a diffusion coefficient of 2.0 µm^2^·s^−1^, complete penetration of the spheroids would take even more than 6 h (Figure 3C). The different diffusion behaviors reflect mostly differences in receptor internalization rates. When simulating penetration at 100 nM for both DARPins, the binding-site barrier was more evident, showing that for 9_26, the simulated diffusion coefficients were not sufficient to reach the core of the spheroids within the 6 h time frame (Figure 3D), whereas for Ec1, only a diffusion coefficient of 10 µm^2^·s^−1^ would achieve full penetration (Figure 3B).

To experimentally validate our model, we investigated DARPin diffusion into tumors and observed similar time-dependent patterns of diffusion into the spheroids for both the EpCAM-targeting DARPin Ec1 and the HER2-targeting DARPin 9_26 at 500 nM (Figure 3E,F). In a matter of hours, DARPins moved to the core of the spheroid, exhibiting the predicted “frozen front” at each time point, consistent with the presence of a binding-site barrier [14]. After 6 h, Ec1 had fully penetrated the spheroid and 9_26 had reached about 90–95%. A lower concentration of 100 nM showed a slower-moving front, and not a generally lower signal (Figure 3E,G). For Ec1, the simulations were most similar to experimental data assuming a diffusion coefficient of 1.0 µm^2^·s^−1^, whereas for 9_26, a simulation employing a diffusion coefficient of 2.0 µm^2^·s^−1^ was most accurate for 500 nM, while at 100 nM, a faster apparent diffusion coefficient would be needed (Figure 3A–D).

These results illustrate the potential for combining simulations of protein delivery in spheroids with optical clearing protocols for an accurate assessment of tumor penetration, allowing us to predict the effects of concentration and affinity on successful delivery, while also serving as an important validation step before moving to more complex microfluidic models.

### 3.3. DARPin Delivery in 3D in a Microfluidic Tumor-on-a-Chip Model

**Figure 4:**
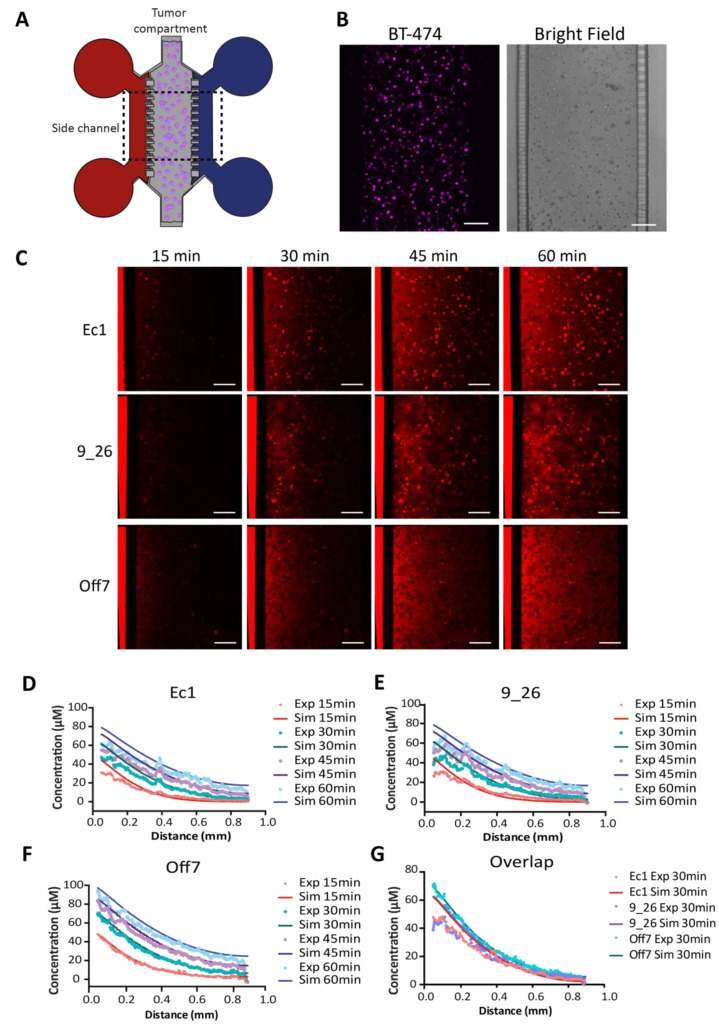
Real-time penetration of DARPins in a tumor-on-a-chip. (**A**) Schematic of the tumor-on-a-chip consisting of a main chamber with BT-474 tumor cells embedded in Matrigel and two side channels connected to medium reservoirs where DARPins (in red) are added to one of the side channels. (**B**) Microscopy images of the BT-474 cells stained with CellTrace violet and a bright-field image of the PDMS chip showing the tumor compartment filled with tumor cells embedded in Matrigel, the side channels, and the microchannels connecting the tumor compartment with the side channels. (**C**) Time points extracted from confocal time-lapse imaging of Alexa Fluor 680-labeled DARPins penetrating in real-time in the tumor tissue in the microfluidic device. Scale bar: 200 µm. (**D**–**F**) Depiction of simulated vs. experimental data for each DARPin and time point. The diffusion coefficient was fitted for Off7 (see main text). (**G**) Overlap of the experimental and simulated data for the three DARPins at *t* = 30 min. Exp—experimental data; Sim—simulation data.

To be able to study DARPin delivery in real-time, we utilized a microfluidic tumor-on-a-chip approach in conjunction with simulations. The ability to compartmentalize allows for precise control over the interface between the 3D tumor tissue and the drug solution, thus enabling an accurate real-time study of tumor penetration in a 3D tumor microenvironment. Here, we focused on transport by diffusion, mimicking tumor regions where little to no interstitial flow is present (Figure 4A). In the microfluidic device, fluorescently labeled DARPins added in the medium reservoirs diffused from the side channels into the main tumor compartment containing BT-474 cells (Figure 4B), and could be followed in real-time during 1 h using confocal microscopy (Figure 4C). The modeling approach for the tumor-on-a-chip can be used to simulate the transport behavior and fit observed behavior in order to extract the apparent diffusion coefficients (Figure 4D–G).

We estimated the diffusion coefficient for DARPins in the tumor tissue by fitting the diffusion profile of the non-binding DARPin Off7 to the simulated results of a range of diffusion coefficients (Figure S5A), yielding an estimate of 55 ± 5 µm^2^ s^−1^, which is much higher than the diffusion constants in spheroids with high cell densities. In both the simulations and experimental data of Ec1 (Figure 4D) and 9_26 (Figure 4E), we observed slower transport into the tissue as a consequence of the predicted DARPin–receptor interactions. To assess the goodness of fit, we calculated the average relative error between the simulated and experimental curves for Off7, 9_26, and Ec1 (Figure S5B–D). The fit was markedly better compared with a model without binding for 9_26 (Figure S5E,F) and Ec1 (Figure S5G,H). Evidently, there was no “frozen front” reflecting a strong binding-site barrier as observed in the spheroids. Instead, the transport behavior of the DARPins in the tumor tissue on-chip, with a lower cell density compared with spheroids, can be best described as hindered diffusion.

**Figure 5:**
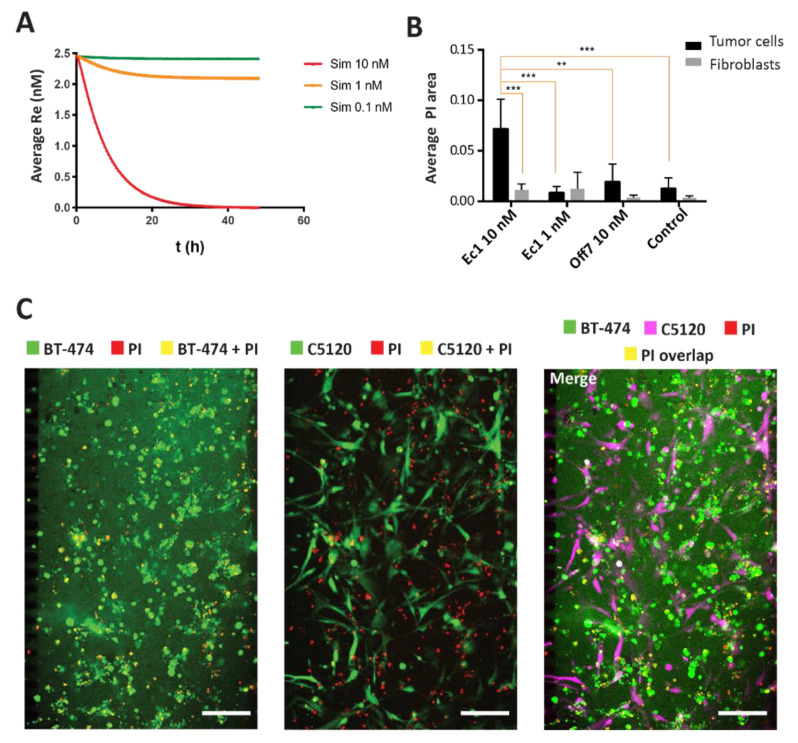
Simulation and experimental verification of Ec1-ETA-mediated tumor cell killing. (**A**) Simulations for Ec1-ETA at concentrations of 0.1 nM, 1 nM, and 10 nM in the model. Toxic effects are approximated in the model through omitting receptor recycling. Overall receptor levels (Re) on the *y*-axis thus serve as a proxy for the overall cell viability. Receptor levels/cell viability is depicted as a function of time integrated over the entire volume of the tumor compartment in the microfluidic device. Re—receptor level reflecting overall viability. (**B**) Quantification of the overlapping area between the PI channel and the cell (tumor or fibroblasts) channels. Error bars reflect SEM. ** means *p* < 0.001, *** means *p* < 0.0001 based on a two-way ANOVA; *n* = 3 (**C**) Microscopy images after 48 h of incubation with 10 nM Ec1-ETA in a co-culture of BT-474 and C5120 cells. The overlapping area between the tumor cells or fibroblasts in green and propidium iodide (PI) in red yields a yellow pseudo color. The scale bars represent 200 µm.

The model was further adapted for the delivery of agents that have toxic effects. Unlike fluorescently labeled agents that are constitutively endocytosed, the receptor regenerated cells that take up toxic agents will eventually be killed, thereby halting receptor regeneration and affecting the kinetics of the protein distribution. In the adapted model that assumed no receptor regeneration as a proxy for cell killing, we predicted the protein diffusion of 0.1, 1, and 10 nM of the targeted fusion toxins (Figure 5A). Given the extremely potent toxic effects of DARPin-toxin fusions [39], we assumed that the remaining free receptor levels reflect the cell viability. The predictions indicated that extensive cell killing would occur only at 10 nM or higher. To compare the simulated data against the experimental data, we tested the activity of the DARPin-ETA fusion toxins in co-cultures of tumor cells with C5120 fibroblasts (1:1 ratio) in the microfluidic model. As our simulation predicted very little toxicity up to 1 nM, the co-cultures were incubated with 1 nM or 10 nM of Ec1-ETA DARPin and 10 nM of Off7 ETA-DARPin as a negative control. At 10 nM, but not at 1 nM, we observed a significant 10-fold increase in cell death, as reflected by the overlapping PI area in BT-474 cells, compared with the controls of Off7-ETA and the controls without DARPin (Figure 5B), consistent with the prediction from our simulations. Furthermore, Ec1-ETA showed a specific killing towards high EpCAM-expressing BT-474 tumor cells, but not towards low EpCAM-expressing fibroblasts in the tumor-on-a-chip (Figure 5B,C).

## 4. Discussion

In this study, we illustrated a hybrid approach that combines the simulation of protein behavior in 3D tumor tissues with an experimental investigation of tumor-targeted DARPins and DARPin-toxin fusion using spheroids and in real-time using a microfluidic tumor-on-a-chip model.

There is still a limited understanding of the dynamics of transport and binding interactions of protein-based targeted agents in complex tumor tissues, which is crucial for designing and evaluating novel therapeutic approaches for optimal target engagement in vivo [43]. We focused on spheroids that represent dense, solid avascular tumors with a high interstitial pressure, which often exhibit a layer-by-layer uptake of protein therapeutics and a binding-site barrier. Alternatively, we used tumors-on-chips to represent scenarios where the cells are more loosely associated and more prone to metastasize.

Mathematical modeling approaches have a long history in drug delivery research, though the large majority has been focused on nanoparticles [44,45]. In contrast, pharmacokinetic/pharmacodynamic modeling has long been used for predicting the overall receptor occupancy of protein-based therapeutics in order to guide dose selection in humans, but these provide little mechanistic insights at the level of the tumor tissue [46]. Very recently, Tang et al. reported an investigation of the dynamics of antibody-target binding in tumor tissue in vivo. Notably, they reported large differences between k_on_ and k_off_, as measured by surface plasmon resonance (SPR) and apparent rates obtained after fitting the model with in vivo data, as well as very large differences in apparent k_off_ rates between stroma-rich and stroma-poor tumor regions. As the model was compartmental, diffusional hindrances were not explicitly modeled in their approach, which could have contributed to the observed slow on- and slow off-rates in the stroma-rich tumor region, as also indicated by the authors [47]. Their approach differs from our approach, where we assumed fixed k_off_ rates and fitted the data according to apparent diffusion coefficients in a spatiotemporal fashion, allowing for an analysis of the gradients.

Our modeling approach is more similar to the approaches followed previously by Thurber and Wittrup, who performed a spatiotemporal investigation of the tumor delivery and binding-site barrier of targeted proteins in vitro [14], and Torres et.al, who performed a theoretical investigation of in vivo protein delivery [28].

In agreement with their findings, we observed a binding-site barrier on the outside of the spheroids when using tumor cells that overexpress membrane molecules such as EpCAM or HER2, and DARPins against these targets. The “frozen front” moves in predictable ways as a function of concentration and time, as has also recently been found in other contexts by us and several other groups who have studied the penetration of binding proteins in spheroids [14,32,48,49]. When fitting the experimental data of the DARPin diffusion in the spheroids, the model predicted diffusion coefficients of 1 µm^2^·s^−1^ for Ec1 and 2 µm^2^·s^−1^ for 9_26. It must be noted that these values rely on reported internalization rates in 2D cultures, which may be different from those in 3D tumor tissues [37,38]. It is noteworthy that the diffusion coefficients we found were low when compared with the 33 µm^2^·s^−1^ reported by Thurber and Wittrup for a single-chain antibody fragment in tumor tissue [14]. Factors that could decrease the diffusion coefficient of the DARPins in the spheroids are related to the density of the collagen or methylcellulose within the spheroids, the cell density and the tightness of the cell–cell junctions [50]. In Thurber and Wittrup’s system, the same spheroid size was obtained with ten times fewer cells, which may have yielded more loosely associated spheroids in which proteins can diffuse faster. In the microfluidic tumor-on-a-chip model, tumor cells grow at a much lower density, explaining the much higher estimated diffusion coefficient of 55 ± 5 μm^2^·s^−1^. This latest value is similar to what would be expected in a relatively dense tumor tissue in vivo for a similarly sized molecule, reflecting, for instance, U87 and HSTS26T tumors grown in vivo in a mouse dorsal chamber [51]. Of note, even though the Matrigel used in this study is mostly a mixture of laminin, collagen IV, and enactin, and reflects a basement membrane [52], while in tumor tissues it is mostly collagen that lowers the diffusion coefficient [51], the transport properties of DARPins in cell-dense tumor tissues can be investigated properly when diffusion coefficients match what is found in vivo and specific binding to matrix components plays a negligible role, as is expected for hydrophilic DARPins with highly specific recognition surfaces.

In contrast with the spheroids model, the tumor-on-a-chip system is a model that allows for the investigation of tumors with lower cell densities, in which the accessibility depends, to a large degree, on the porosity of the ECM that causes diffusional hindrance [51]. In these kinds of tumors, the proteins will face diffusion through the extracellular matrix as a main challenge, a notorious problem especially in pancreatic adenocarcinomas [53]. Hence, the proteins will experience a hindered diffusion through the 3D matrix, which is in line with the simulations of our mathematical model, and which is distinct from the frozen front observed by us and others in spheroids. The developed model was also employed to simulate the transport of a DARPin fused to a toxin, which specifically binds and selectively kills tumor cells in the microfluidic device. This combined approach can lead us to better study the specific delivery of protein therapies in a complex 3D system, while also predicting the minimally needed concentration for optimal therapeutic effects. Further applications of our approach can bring us closer towards rational therapeutic protein design. Combined with developments in high-throughput production and approaches towards de novo protein design with desired properties [54], faster in vitro testing using microfluidic organ-on-chip technologies could help with predicting and developing novel and more powerful therapies that can be tested more rapidly and be faster translated to the patient [55].

## 5. Conclusions

To summarize, the key achievement of this study is the illustration of the power of a hybrid approach that combines the use of biomimetic in vitro tumor models with mathematical models to study and predict protein delivery and activity in complex 3D systems. Our hybrid approach has great potential for the development of novel potential protein therapies, as it in part sidesteps the extrapolation challenges from 2D or animal models to complex human physiology by efficiently combining in silico approaches with biologically relevant in vitro test systems.

## Figures and Tables

**Table 1:** Parameters used in the mathematical model for describing protein delivery in multicellular tumor spheroids and in a microfluidic tumor-on-a-chip.

Parameter	Value and Units	Reference
Void fraction tumor-on-a-chip (ε)	0.80	Experimental ^a^
Void fraction spheroids (ε)	0.15	[14]
Re_0_ tumor-on-a-chip (BT-474)	HER2: 2.18 nm EpCAM: 2.45 nm	Calculated ^b^
Re_0_ spheroids (BT-474)	HER2: 234 nm EpCAM: 263 nm	Calculated ^d^
k_on_	HER2-binding DARPin 9_26: 7.38 × 10^4^ m^−1^·s^−1^ EpCAM-binding DARPin Ec1: 3.65 × 10^5^ m^−1^·s^−1^	[35,36]
k_off_	HER2-binding DARPin 9_26: 0.1 × 10^−3^ s^−1^ EpCAM-binding DARPin Ec1: 3.65 × 10^5^ s^−1^	[35,36]
k_e_	HER2: 1.67 × 10^−4^ s^−1^ EpCAM: 3.46 × 10^−5^ s^−1^	[37,38]
Diffusion coefficient D_Water_ (DARPin)	164 µm^2^·s^−1^	Estimated ^c^

^a^ The void fraction was estimated by determining the cell occupancy in the chip from a threshold applied to confocal images of the cell channel (CellTrace yellow-stained BT-474 cells) and subtracting the percentage of positive pixels in the images from the total gel area. ^b^ Calculated based on quantified values for the number of receptors per cell and number of cells in the microfluidic chip or in the spheroids; ^c^ The diffusion coefficient of DARPins in water was estimated based on the Stokes–Einstein law by taking into account the hydrodynamic radius of DARPins, as reported previously [30]. ^d^ In the spheroid model, diffusion coefficients were varied and evaluated against experimental data.

## Data Availability

The data that support the findings of this study are contained within the article and supplementary materials. Raw data are available from the corresponding author upon reasonable request.

## Supplementary Materials

The following are available online at https://www.mdpi.com/article/10.3390/cancers13102461/s1, Figure S1: Purified DARPins and DARPin-toxin fusions, Figure S2: Flow cytometry-based quantification of receptor density in different cell lines, Figure S3: Binding of DARPins to BT-474 cells, Figure S4: Schematic representation of the input variables and outcomes determined by the binding-diffusion mathematical model, Figure S5: Fitting of the mathematical model to the experimental data, Figure S6: Long term viability assay after incubation with DARPin-toxin fusions, Supplementary methods, 1: quantification of cell death in tumor-on-a-chip system, 2: analysis of spheroid penetration.
