# Supplementary material for: A Hybrid In Silico and Tumor-on-a-Chip Approach to Model Targeted Protein Behavior in 3D Microenvironments
Source: Cancers (Basel). 2021 May 18;13(10):2461. doi: 10.3390/cancers13102461 (PMC8158470; doi:10.3390/cancers13102461)
Supplement: Supplementary file 1 [file cancers-13-02461-s001.zip › cancers-1214023-supplementary.pdf]

cancers

Supplementary Materials: A Hybrid in Silico and Tumor-on-a-Chip Approach to Model Targeted Protein Behavior in 3D Microenvironments

Valentina Palacio-Castañeda, Simon Dumas, Philipp Albrecht, Thijmen J. Wijgers, Stéphanie Descroix and Wouter P.R. Verdurmen

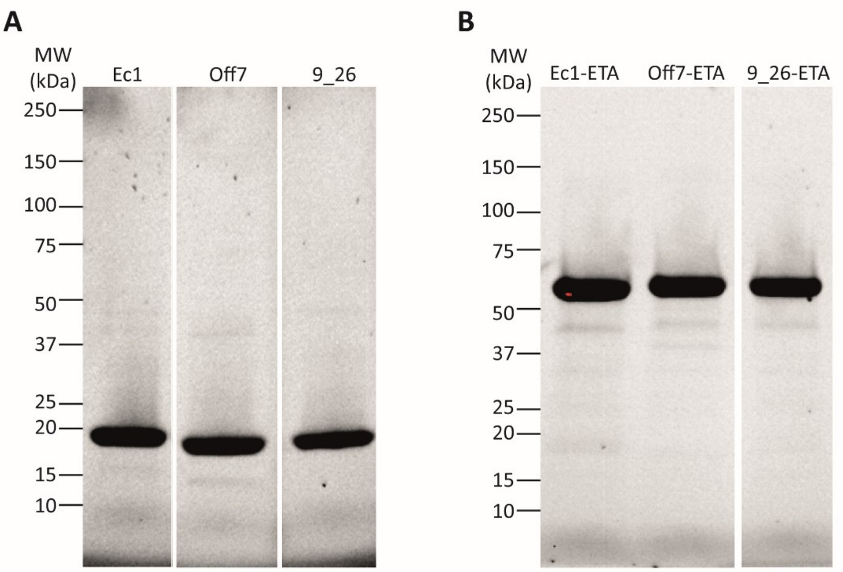

Figure S1. Purified DARPins and DARPin-toxin fusions**.** (**A**) SDS-PAGE showing the purified EpCAM-binding DARPin Ec1, the HER2-binding DARPin 9_26 and the non-binding control Off7. (**B**) SDS-PAGE showing purified the EpCAM-binding DARPin-toxin fusion Ec1-ETA, the HER2-binding DARPin-toxin fusion 9_26-ETA and non-binding control DARPin-toxin fusion Off7-ETA.

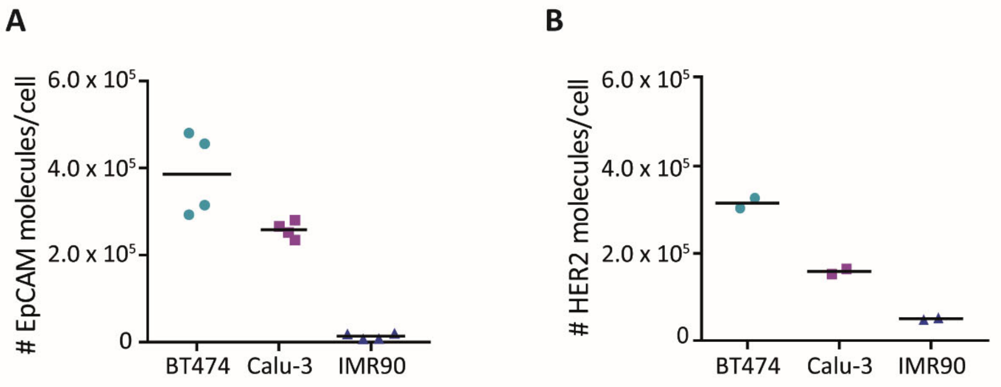

Figure S2. Flow cytometry-based quantification of receptor density in different cell lines (**A**) Quantification of EpCAM molecules per cell. Horizontal bar depicts mean. (**B**) Quantification of HER2 molecules per cell. Horizontal bar depicts mean.

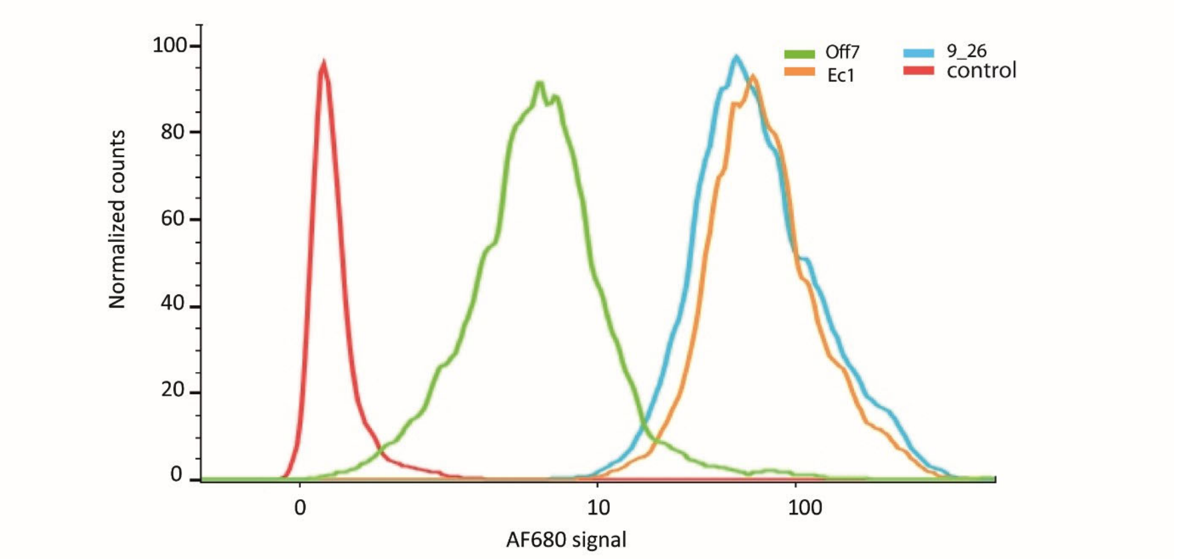

Figure S3. Binding of DARPins to BT-474 cells. Representative flow cytometry histogram of BT-474 cells incubated with DARPin-Alexa Fluor 680 conjugates for 1 h at 37 °C.

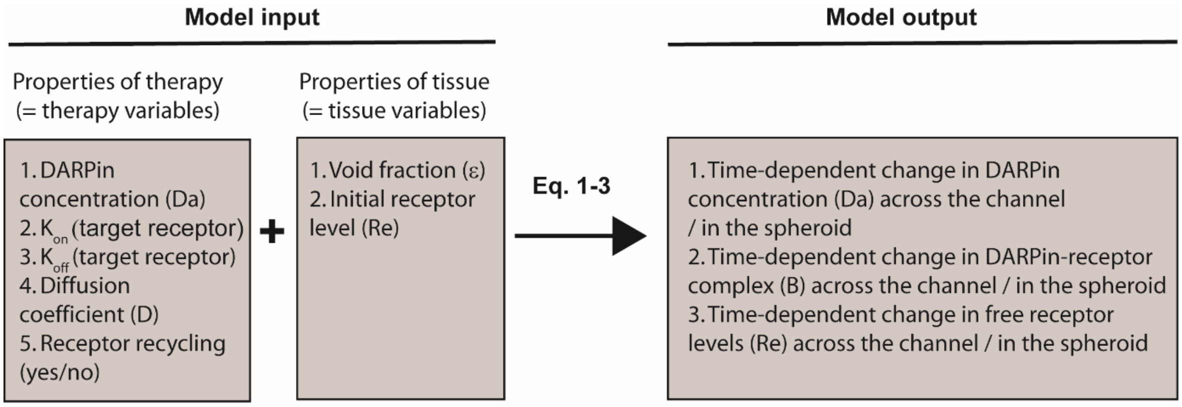

Figure S4. Schematic representation of the input variables and outcomes determined by the binding-diffusion mathematical model. Relation between model input and model output is schematically depicted. Eq. 1-3 are given in full in section 2.13 of the materials and methods.

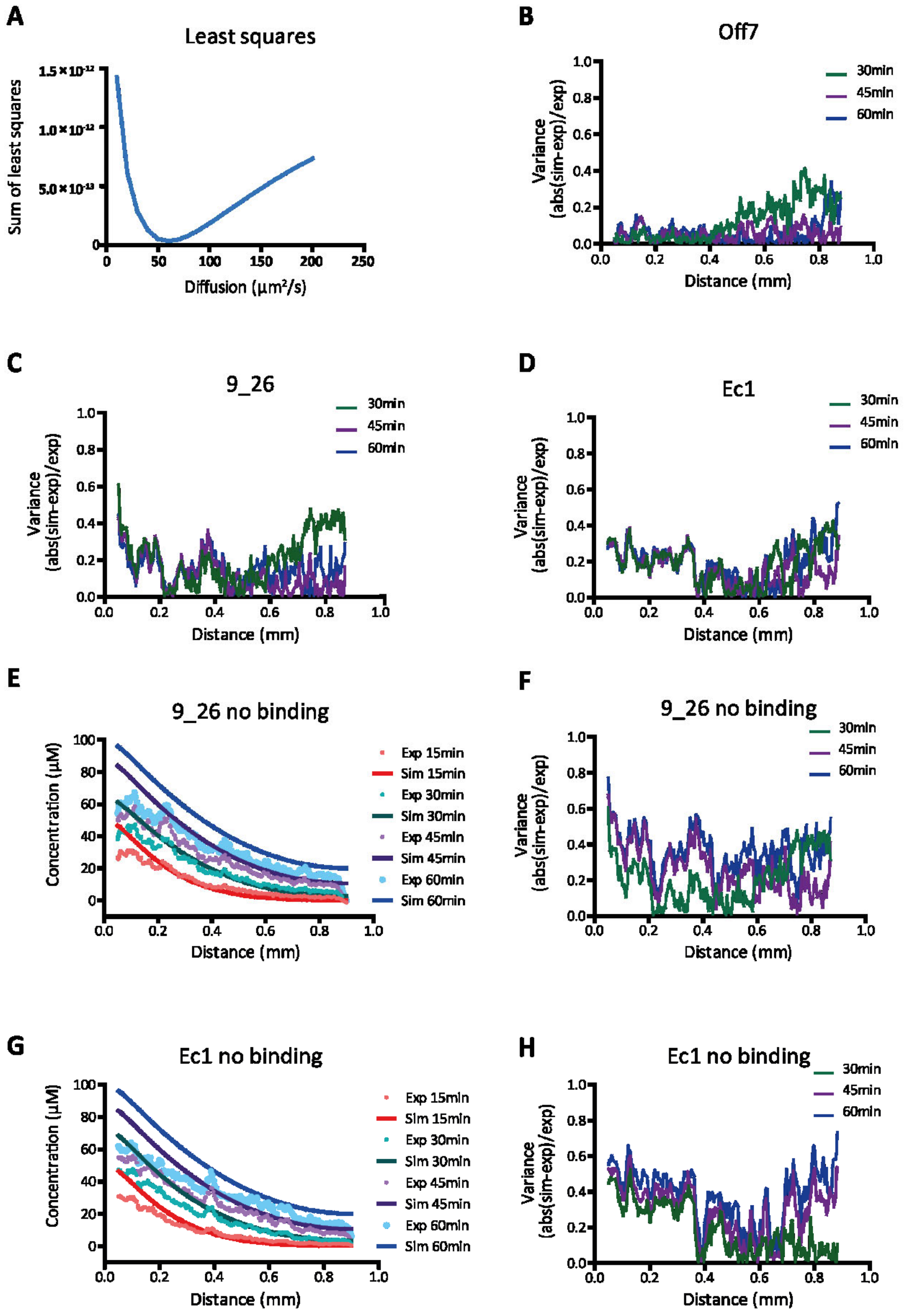

Figure S5. Fitting of the mathematical model to the experimental data. (**A**) Optimization using the least squares method to find the diffusion coefficient of Off7. Simulations were performed with different diffusion coefficients ranging from 10 to 200 μm^2/s. For each one of them, we calculated a parameter, called a sum of least-squares, to estimate how close the

experimental and simulated profiles are close to each other. The profiles are measured in the tumor-on-chip main chamber at a height of 200 μm both on experiments and simulations. A least-squares is defined for each space and time point as 〖√(exp-simu)〗^2, after which they are added together to provide a single parameter for each diffusion coefficient. Figure S5A shows these sums of least-squares for different diffusion coefficients. A minimal sum of least-squares indicates a good fitting of the simulation with experimental profiles. Based on two experiments, we obtained the best fitting with 55 μm2/s. For this value, the average relative error (%) between experimental and simulated data was calculated. For each x, the Variance = abs(sim-exp)/exp was calculated for Off7 (**B**), 9_26 (**C**) and Ec1 (**D**). (**E**–**H**) For comparison, similar simulations and calculations of relative error rates from a model without the inclusion of a term for the binding of the DARPins to the receptor were performed.

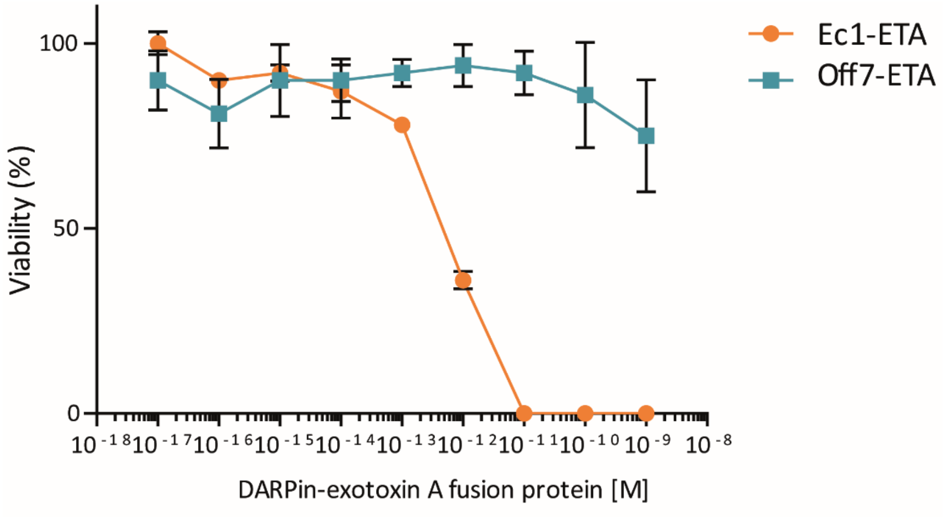

Figure S6. Long term viability assay after incubation with DARPin-toxin fusions**.** Percentage of viability of Calu-3 cells 5 days after incubation with different concentrations of the EpCAM-binding DARPin-toxin fusion Ec1-ETA and the non-binding control DARPin-toxin fusion Off7-ETA. Viability was measured with the resazurin assay. Error bars indicate SEM, *n* = 3.

Supplementary Methods

1. Quantification of cell death in tumor-on-a-chip system

The macro below was used to quantify the overlap area between the propidium iodide (PI) signal and the cell area in order to determine percentage of cell death.

For Fibroblasts

```
from ij import IJ, ImagePlus, WindowManager
from ij.gui import Roi, PointRoi
from ij.process import ImageProcessor
from ij.plugin.filter import ThresholdToSelection, GaussianBlur
piImage = WindowManager.getImage("PI.tif")
fibroblastsImage = WindowManager.getImage("Fibroblasts.tif")
#Apply Gaussian Blur
gaussianBlurrer = GaussianBlur()
gaussianBlurrer.blurGaussian (piImage.getProcessor(), 2)
gaussianBlurrer.blurGaussian (fibroblastsImage.getProcessor(), 2)
```

# Applies threshold

```
piImage.getProcessor().setThreshold(75, 255, ImageProcessor.NO_LUT_UPDATE)
fibroblastsImage.getProcessor().setThreshold(10, 255, ImageProcessor.NO_LUT_UP-
DATE)
```

# Converts the thresholds into ROIs

```
piRoi = ThresholdToSelection.run(piImage)
fibroblastsRoi = ThresholdToSelection.run(fibroblastsImage)
piImage.setRoi (piRoi)
fibroblastsImage.setRoi (fibroblastsRoi)
```

# Gets all points inside both ROIs, and only keeps overlapping ones piRoiPoints = piRoi.getContainedPoints()

```
fibroblastsRoiPoints = fibroblastsRoi.getContainedPoints()
overlappingPoints = list(set(piRoiPoints) & set(fibroblastsRoiPoints))
```

# Creates ROI containing overlapping points

```
overlappingRoi = PointRoi()
for p in overlappingPoints:
overlappingRoi.addPoint(p.x, p.y)
```

#Create duplicate images and show the overlapping ROI

```
duplicatedPiImage = piImage.duplicate ()
duplicatedPiImage.show ()
duplicatedPiImage.setRoi (overlappingRoi)
duplicatedFibroblastsImage = fibroblastsImage.duplicate ()
duplicatedFibroblastsImage.show ()
duplicatedFibroblastsImage.setRoi (overlappingRoi)
```

# Measures area of both ROIs and calculates the percentage overlapping piArea = piRoi.getStatistics().area

```
fibroblastsArea = fibroblastsRoi.getStatistics().area
overlappingArea = overlappingRoi.getStatistics().area
overlappingRatio = overlappingArea / piArea
print (piArea, overlappingArea, overlappingRatio)
```

For BT-474

```
from ij import IJ, ImagePlus, WindowManager
from ij.gui import Roi, PointRoi
from ij.process import ImageProcessor
from ij.plugin.filter import ThresholdToSelection, GaussianBlur
piImage = WindowManager.getImage("PI.tif")
bt474Image = WindowManager.getImage("BT474.tif")
```

#Apply Gaussian Blur

```
gaussianBlurrer = GaussianBlur()
gaussianBlurrer.blurGaussian (piImage.getProcessor(), 2)
gaussianBlurrer.blurGaussian (bt474Image.getProcessor(), 2)
```

# Applies threshold

```
piImage.getProcessor().setThreshold(75, 255, ImageProcessor.NO_LUT_UPDATE)
bt474Image.getProcessor().setThreshold(30, 255, ImageProcessor.NO_LUT_UPDATE)
```

# Converts the thresholds into ROIs

```
piRoi = ThresholdToSelection.run(piImage)
bt474Roi = ThresholdToSelection.run(bt474Image)
```

piImage.setRoi (piRoi)
bt474Image.setRoi (bt474Roi)

# Gets all points inside both ROIs, and only keeps overlapping ones piRoiPoints = piRoi.getContainedPoints()

bt474RoiPoints = bt474Roi.getContainedPoints()
overlappingPoints = list(set(piRoiPoints) & set(bt474RoiPoints))

# Creates ROI containing overlapping points

overlappingRoi = PointRoi()
for p in overlappingPoints:
overlappingRoi.addPoint(p.x, p.y)

#Create duplicate images and show the overlapping ROI

duplicatedPiImage = piImage.duplicate ()
duplicatedPiImage.show ()
duplicatedPiImage.setRoi (overlappingRoi)
duplicatedBt474Image = bt474Image.duplicate ()
duplicatedBt474Image.show ()
duplicatedBt474Image.setRoi (overlappingRoi)

# Measures area of both ROIs and calculates the percentage overlapping piArea = piRoi.getStatistics().area

bt474Area = bt474Roi.getStatistics().area
overlappingArea = overlappingRoi.getStatistics().area
overlappingRatio = overlappingArea / piArea
print (piArea, bt474Area, overlappingArea, overlappingRatio)

2. Analysis of spheroid penetration

The python script below was used to analyse penetration of DARPins into cleared spheroids *from ij import IJ, ImagePlus*

from ij.io import OpenDialog
from ij.gui import GenericDialog, WaitForUserDialog, Roi, Line, ProfilePlot from ij.plugin.filter import GaussianBlur
from ij.io import RoiEncoder
from loci.plugins import BF
from loci.plugins.in import ImporterOptions
from java.awt import Point
from math import ceil
import os, re, csv
def getLifFileImportOptions(path):
options = ImporterOptions()
options.setId(path)
options.setAutoscale(True)
options.setColorMode(ImporterOptions.COLOR_MODE_GRAYSCALE)
options.setConcatenate(False)
options.setCrop(False)
options.setFirstTime(True)
options.setGroupFiles(False)
options.setMustGroup(False)
options.setOpenAllSeries(True)
options.setROIsMode(ImporterOptions.ROIS_MODE_MANAGER)
options.setShowMetadata(False)

options.setShowOMEXML(False)
options.setShowROIs(False)
options.setSpecifyRanges(False)
options.setSplitChannels(True)
options.setSplitFocalPlanes(False)
options.setSplitTimepoints(False)
options.setStackFormat(ImporterOptions.VIEW_HYPERSTACK)
options.setStackOrder(ImporterOptions.ORDER_XYCZT)
options.setStitchTiles(False)
options.setSwapDimensions(False)
options.setUngroupFiles(False)
options.setUpgradeCheck(False)
options.setVirtual(True)
options.setWindowless(False)
return options
def getSingleImageFromLifFile(path):
options = getLifFileImportOptions(path)
options.setOpenAllSeries(False)
return BF.openImagePlus(options)
def readImagesFromLifFile(path):
options = getLifFileImportOptions(path)
return BF.openImagePlus(options)
def getChannelInfo(path):
images = getSingleImageFromLifFile(path)
channelCount = len(images)
channelNames = ["Channel " + str(i) for i in xrange(channelCount)]
dialog = GenericDialog("Select channels")
for index, name in enumerate(channelNames):
channelImageProcessor = images[index].duplicate().getProcessor().resize(100) channelImage = ImagePlus(name, channelImageProcessor)
dialog.addMessage(name)
dialog.addImage(channelImage)
dialog.addChoice("Channel to analyse", channelNames, channelNames[0]) *dialog.showDialog()*
if dialog.wasCanceled():
return
channelToAnalyzeIndex = dialog.getNextChoiceIndex()
return channelCount, channelToAnalyzeIndex
lif_path = OpenDialog("Select lif file to analyse").getPath()
channelInfo = getChannelInfo(lif_path)
if channelInfo is None:
quit()
channelCount, channelToAnalyzeIndex = channelInfo
channelsToMerge = list(xrange(channelCount))
images = readImagesFromLifFile(lif_path)
imageCount = len(images) / channelCount
sampleNameRegex = re.compile(r"- (.*?) - C=\d$")
outputDirectory = os.path.splitext(lif_path)[0]
outputCSVDirectory = os.path.join(outputDirectory, "Results") outputRoiDirectory = os.path.join(outputDirectory, "ROIs") outputSliceDirectory = os.path.join(outputDirectory, "Slices") if os.path.exists(outputDirectory):
quit()
os.mkdir(outputDirectory)
os.mkdir(outputCSVDirectory)

```
os.mkdir(outputRoiDirectory)
os.mkdir(outputSliceDirectory)
def getMaxAndDistance(profileData, scale):
xValues, yValues = zip(*profileData)
if not xValues[1] == 1:
scale = 1 / scale
maxValue = max(yValues)
maxIndex = yValues.index(maxValue)
targetValue = maxValue / 2
leftIndex = 0
while True:
value = yValues[leftIndex]
if value > targetValue:
break
elif leftIndex == maxIndex:
break
leftIndex += 1
if abs(yValues[leftIndex + 1] - targetValue) < abs(yValues[leftIndex] - targetValue):
leftIndex += 1
rightIndex = len(yValues) - 1
while True:
value = yValues[rightIndex]
if value > targetValue:
break
elif rightIndex == maxIndex:
break
rightIndex -= 1
if abs(yValues[rightIndex - 1] - targetValue) < abs(yValues[rightIndex] - targetValue):
rightIndex -= 1
leftDistance = xValues[leftIndex]
rightDistance = xValues[rightIndex]
distance = rightDistance - leftDistance
radius = xValues[-1] - leftDistance
return maxValue, distance * scale, radius * scale
 def formatNumber(n):
return str(n).replace(".", ",")
for i in xrange(imageCount):
offset = i * channelCount
sampleName = sampleNameRegex.search(images[offset].getTitle()).group(1) if sam-
pleName.lower() == "settings":
continue
imageToAnalyze = images[channelToAnalyzeIndex + offset].duplicate() roiSelection-
Image = imageToAnalyze.duplicate()
IJ.run(roiSelectionImage, "Fire", "")
roiSelectionImage.show()
dialog = WaitForUserDialog("Please encircle the spheroid, then click OK to con-
tinue.") dialog.show()
micronPerPixel = imageToAnalyze.getLocalCalibration().pixelWidth roiSelectionIm-
age.hide()
roi = roiSelectionImage.getRoi()
if not roi:
quit()
sliceIndex = roiSelectionImage.getCurrentSlice()
```

selectedSlice = ImagePlus("", imageToAnalyze.getStack().getProcessor(sliceIndex)) *selectedSliceNotBlurred = selectedSlice.duplicate() 48*

gaussianBlurrer = GaussianBlur()

gaussianBlurrer.blurGaussian(selectedSlice.getProcessor(), 10)

IJ.saveAsTiff(selectedSliceNotBlurred, os.path.join(outputSliceDirectory, sampleName)) *IJ.saveAsTiff(selectedSlice, os.path.join(outputSliceDirectory, sampleName + "_blurred")) RoiEncoder.save(roi, os.path.join(outputRoiDirectory, sampleName + "_ROI_.roi"))* centerPoint = Point(int(roi.getBounds().getCenterX()), int(roi.getBounds().getCenterY())) roiPoints = map(lambda (x, y): Point(x, y), zip(roi.getPolygon().xpoints, roi.getPolygon().ypoints))

numberOfLines = 360

profileDatas = []

measurements = []

for i in xrange(numberOfLines):

endPoint = roiPoints[len(roiPoints) * i / numberOfLines]

line = Line(endPoint.x, endPoint.y, centerPoint.x, centerPoint.y)

selectedSlice.setRoi(line)

profilePlot = ProfilePlot(selectedSlice)

profileData = zip(profilePlot.getPlot().getXValues(), profilePlot.profile) *profileDatas.append(profileData)*

measurement = getMaxAndDistance(profileData, micronPerPixel)

measurements.append(measurement)

with open(os.path.join(outputCSVDirectory, sampleName + ".csv"), "wb") as csvFile: csvFile.write("sep=;\n")

writer = csv.writer(csvFile, delimiter=';')

sumMaxIntensity = 0

sumDistance = 0

sumRadius = 0

for i in measurements:

sumMaxIntensity += i[0]

sumDistance += i[1]

sumRadius += i[2]

writer.writerow(["Max intensity", "Distance", "Radius", "", "Mean max intensity", "Mean distance", "Mean radius", "Mean diameter"]) writer.writerow([formatNumber(measurements[0][0]), formatNumber(measurements[0][1]), formatNumber(measurements[0][2]), "", formatNumber(sumMaxIntensity / numberOfLines), formatNumber(sumDistance / numberOfLines), formatNumber(sumRadius / numberOfLines), formatNumber(sumRadius * 2 / numberOfLines)])

for i in measurements[1:]:

writer.writerow([formatNumber(i[0]), formatNumber(i[1]), formatNumber(i[2])]) *with open(os.path.join(outputCSVDirectory, sampleName + "_RAW.csv"), "wb") as csvFile:* csvFile.write("sep=;\n")

writer = csv.writer(csvFile, delimiter=';')

headers = [["Line " + str(x + 1), "", ""] for x in xrange(numberOfLines)]

headers = [item for subheaders in headers for item in subheaders]

writer.writerow(headers)

writer.writerow(["Distance", "Intensity", ""] * numberOfLines) max_length = max([len(profileData) for profileData in profileDatas]) for i in xrange(max_length):

row = []

for l in xrange(numberOfLines):

try:

row.extend([formatNumber(profileDatas[l][i][0]), formatNumber(profileDatas[l][i][1])]) *except:*

```
row.extend(["", ""])
row.append("")
writer.writerow(row)
```
